# Quantifying Proportional Variability

**DOI:** 10.1371/journal.pone.0084074

**Published:** 2013-12-30

**Authors:** Joel P. Heath, Peter Borowski

**Affiliations:** Department of Mathematics, University of British Columbia, Vancouver, British Columbia, Canada; Queen's University Belfast, United Kingdom

## Abstract

Real quantities can undergo such a wide variety of dynamics that the mean is often a meaningless reference point for measuring variability. Despite their widespread application, techniques like the Coefficient of Variation are not truly proportional and exhibit pathological properties. The non-parametric measure Proportional Variability (PV) [1] resolves these issues and provides a robust way to summarize and compare variation in quantities exhibiting diverse dynamical behaviour. Instead of being based on deviation from an average value, variation is simply quantified by comparing the numbers to each other, requiring no assumptions about central tendency or underlying statistical distributions. While PV has been introduced before and has already been applied in various contexts to population dynamics, here we present a deeper analysis of this new measure, derive analytical expressions for the PV of several general distributions and present new comparisons with the Coefficient of Variation, demonstrating cases in which PV is the more favorable measure. We show that PV provides an easily interpretable approach for measuring and comparing variation that can be generally applied throughout the sciences, from contexts ranging from stock market stability to climate variation.

## Introduction

Understanding the variability of a quantity is a fundamental concept. The concept is generally considered intuitive, and techniques for measuring variability are rarely given a second thought, despite well established pathological issues [2]–[4]. Variation is typically based on calculating the average deviation from the mean. This of course assumes that the mean is a meaningful starting point for measuring variation. Given the central limit theorem, this may be a reasonable assumption in terms of statistical sampling; however, in terms of dynamics, real quantities rarely fluctuate about a central point and can exhibit a diverse spectrum of dynamics ranging from simple oscillation to chaos and noise [5]. There is nothing normal (Gaussian) about these dynamics and the mean can therefore be a misleading reference point for measuring variation. A common ground for measuring and comparing overall variation among quantities undergoing different dynamics requires a framework that is not secondarily based on a measure of central tendency.

There has been extensive confusion in several disciplines about the appropriate way to measure variation [2]–[4], [6], [7]. Given the standard deviation is related to the mean [8], the most commonly used and advocated technique for measuring variation on a proportional scale is the Coefficient of Variation (CV), computed as the standard deviation divided by the mean. Despite its widespread application, it has many pathological properties that can lead to inappropriate interpretation of results. In particular, it is not a truly proportional measure of variability, as it is not bounded by an upper value of 1. In contexts like population ecology, rare events are known to severely bias the CV, whereas a robust measure of variation should not rely on subjective decisions about what is rare and common nor involve inappropriately weighting or excluding data [9]. In the context of population dynamics, Heath [1] developed a simple solution to this issue: simply compare the numbers to each other rather than to an average. Using numerical simulations, it was shown that this technique is not biased by rare events or non-Gaussian dynamics, allows more accurate estimation of long term variability from short term data sets, and allows robust summarization and comparison of variability (or inversely, stability) among quantities undergoing very different dynamics. The present research uses mathematical proofs and an analytical approach to demonstrate desirable properties of Proportional Variability (PV), resolving important standing issues and providing a general replacement for the Coefficient of Variation. Variability is one of the most fundamental concepts in the sciences, and is particularly important for understanding contemporary issues including economic and environmental change. PV provides an intuitive and robust common ground for measuring and comparing variation on a proportional scale, and a new paradigm for concepts of variability.

## Methods

Proportional Variability (PV) is based on a ratio comparison of all numbers. For a given data set of 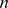 non-negative points 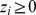, there will be 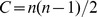 unique pairwise combinations of 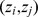, for which we calculate the relative difference 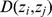. PV is therefore defined as:
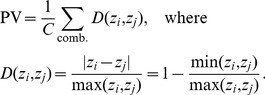
(1)


Unlike CV, the domain of PV is on a truly proportional scale 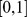. Like the CV, this provides a summary of variation where the chronology of the data is irrelevant, and PV effectively considers variation at all scales or time lags. However, by ordering the data in a sequence of increasing magnitude, we can demonstrate some very desirable characteristics of a truly proportional measure of variability. Of course, if the data is constant, PV = 0, and the series is a simple horizontal line. If there is variability, the ordered series will be increasing, the steepness depending on the extent of variability. If the ordered series is linearly increasing, i.e., as an arithmetic sequence, and in the case the starting element of this sequence is zero, it is simple to prove that PV will equal exactly 0.5 independent of sample size 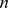:
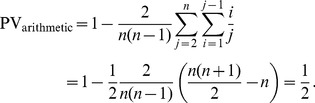
(2)


If variability as measured by PV is greater than 0.5, the values in the ordered series are increasing non-linearly. At the extreme, if variation increases exponentially with time as an ordered geometric series (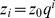 with the common ratio 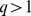), PV will approach a value of 1 as the sample size goes to infinity:
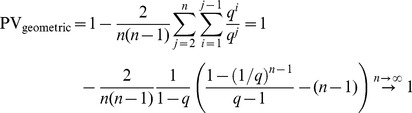
(3)for 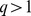, and a similar proof is true for 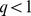. This demonstrates very desirable and intuitive attributes of a proportional measure of variability. In contrast, CV has a domain that is theoretically 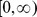, rather inappropriate for a measure interpreted as being proportional.

If a system is normal (Gaussian), the Coefficient of Variation (and many other metrics based upon a measure of central tendency), is particularly excellent at providing useful first and second order summaries of the data (e.g. mean and standard deviation, respectively). By considering continuous probability distributions, we will demonstrate that PV behaves quantitatively similar to the CV under these conditions. This is desirable, but as we describe, the assumptions required for using CV will never be fully met by any systems of real (positive) quantities. We demonstrate that unlike CV, PV can be calculated for continuous distributions with an undefined mean. We conclude by considering bimodal distributions (e.g. simple oscillations), and show that PV is a cure for the pathological properties of the CV when otherwise stable populations exhibit rare events.

## Results

For a continuous probability distribution 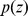 of a non-negative real variable 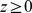, PV can be computed in the following way:
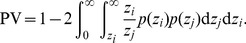
(4)


In this section, we will analytically solve Eq. 4 for different continuous probability distributions, but first, we will consider the Gaussian or 'normal' distribution, where we solve Eq. 4 numerically. While rarely considered in practice, one issue with assuming data conform to a normal continuous distribution is the implication that some, albeit perhaps a small amount of numbers, can be negative. This is obviously an incorrect assumption for real positive quantities such as population abundances or temperature (the latter being analyzed in *Kelvins*). PV does not require this false assumption and is only appropriate for positive quantities. Therefore, to avoid contributions from negative values, we center the mean of the normal distributions at least two standard deviations in the positive domain, set the distribution equal to zero at negative values and renormalize the remaining part accordingly.

**Figure 1:**
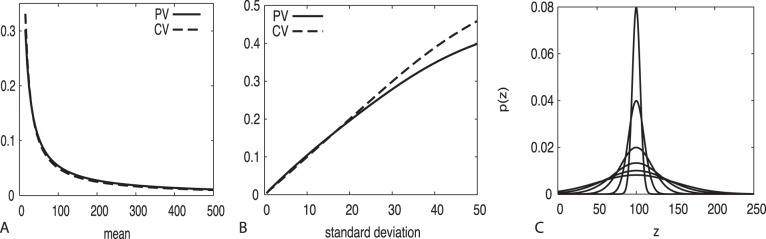
Proportional Variability (PV) and the Coefficient of Variation (CV) correspond closely and quantitatively over a large range of parameters for the Gaussian distribution. Before truncating negative numbers and renormalizing, (A) standard deviation = 5, and (B) stable mean = 100 with increasing standard deviation (as per C, 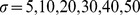). Both CV and PV have been obtained by numerically solving the defining integral equations.

PV behaves quantitatively similar to CV across a wide range of distributions and qualitatively similar for very fat distributions (Figure 1, i.e., CV is larger for fat distributions as extreme values in the tails of the distribution are given substantially more weight due to deviations from the mean being squared in calculating the standard deviation). This indicates PV is a useful replacement for the CV under normal conditions. Because PV is independent of the mean, it also allows statistical analysis of variation vs. the mean, an inappropriate analysis for the CV.

As another test, we compute CV and PV for a uniform distribution centered at 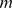 with width 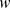 (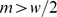). Equation 4 leads to:
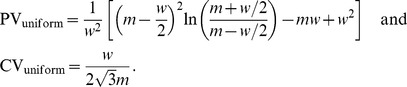
(5)


These expressions for PV and CV are very close to each other for 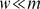, though they differ close to 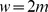 with 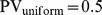 and 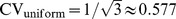. PV is therefore quantitatively similar to CV under normal conditions, providing an appropriate replacement. In a further example, we calculate CV and PV for the exponential distribution
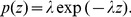
(6)


After some algebra, it turns out that both 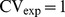 and 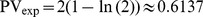, are independent of the decay constant 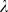 in this case.

PV has the advantage compared to CV and other mean-based metrics, that it can be computed even when a mean is not defined. The Pareto distribution (e.g. [10]) is useful for describing many observable phenomenon such as the skewed distribution of wealth. It is defined as:
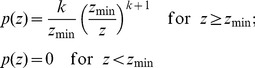
(7)with the two parameters scale 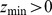 and shape 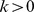. For the Pareto distribution, a mean is only defined if the shape parameter 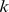 is larger than one, and a second moment (including CV) is only defined for 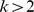. PV is a first order measure of variability and can be computed using Equation 4 for all 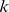 to 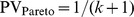, independent of the scale parameter 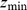.

The mean is also not defined for the heavy-tailed Cauchy-Lorentz (CL) distribution (e.g. [10])
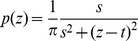
(8)which is described by a scale 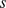 and location parameter 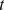. We restrict our analysis to positive CL distributions, considering the positive half of the distribution with a location parameter 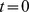 (and an according normalization factor of two). The CV cannot be calculated for the CL distribution. Interestingly, for this special case 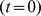, the definite integrals in Eq. 4 can be solved analytically, leading to the result 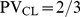, which is independent of the scale parameter 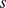. This indicates that contributions near 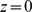 and large 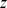 perfectly balance each other out as the scale parameter changes for this distribution. Heath [1] demonstrated numerically that PV is much more appropriate when rare events occur, which is the case for heavy-tailed Cauchy-Lorentz distributions. This is because by comparing each number to every other number, rare events are evaluated in direct relation to their frequency (i.e., how rare they are). PV therefore solves the problem of rare events and allows calculating variability without inappropriate decisions to include outliers or not.

Bimodal distributions offer another intuitive way to compare measures of variability for rare events or other mixed distributions such as those produced by simple oscillatory dynamics. As a clear demonstration, we analyse discrete distributions with 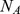 and 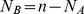 counts at only two magnitudes, 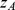 and 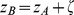. In this case, with 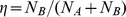


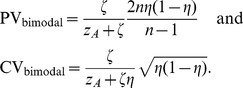
(9)


**Figure 2:**
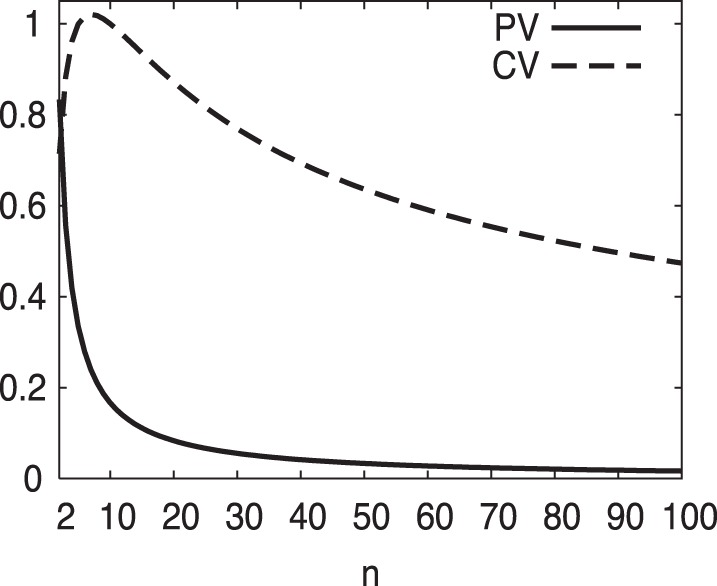
For a quantity which is stable at all time intervals except for a single rare event, CV displays a pathological increase in magnitude with an increase in sample size 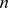 before slowly decreasing. PV appropriately declines monotonically to zero as the frequency of the rare event decreases with increasing sample size for this otherwise stable quantity. In this example 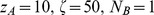.

The first notable observation here is that when exchanging 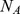 and 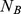 (i.e., replacing 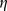 with 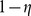 while keeping sample size 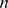 constant), PV behaves symmetrically, maintaining the same value, whereas CV changes in magnitude. The difference between PV and CV (and indeed other central tendency based measures) becomes very obvious when considering large deviations 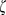 between the two magnitudes (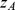 and 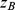) and large differences between the two counts (i.e., 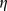 close to zero or to one). The latter is the regime of rare events. In Figure 2, we chose a particularly demonstrative example, a perfectly stable population with all elements at the same common value and only a single rare event. Increasing the sample size 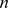 while maintaining a single rare event (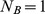) means that the proportion of rare events will be decreasing monotonically due to increasing counts of the common value. As seen in Figure 2, PV decreases monotonically with sample size as expected and is appropriate, however CV actually increases initially for low sample sizes before beginning a slow decrease. This characteristic of CV is particularly pathological, and an inappropriate description of variability for this quantity which is otherwise always stable.

A well-known measure of distribution that behaves similarly to PV for the case of a bimodal distribution is the relative mean difference (RMD) or related Gini coefficient (defined as half of RMD – e.g. [11]). For the remaining distributions discussed in the present report, expressions for RMD can be found elsewhere (e.g. [12]). RMD (and therefore Gini coefficient) is based on the mean, which is why RMD can not be computed for mean-less distributions like Cauchy-Lorentz or Pareto with 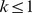.

## Discussion

This treatment demonstrates the wide applicability and robustness of Proportional Variability (PV). While CV is only appropriate for normal distributions, PV behaves the same as CV under these conditions, but also functions as desired for all other possible forms of dynamics. This makes PV particularly appealing for summarizing and comparing variability in data that can undergo a wide variety of dynamics, such as in non-linear economic, physical and biological systems [5]. Heath [1] also numerically demonstrated that PV behaves the same as the spectral exponent (a useful gold standard) when used to evaluate more time more variation (reddened spectrum), whereas CV inappropriately suggests spectral reddening in stationary time series. Furthermore, PV allows substantially more accurate estimates of known long term variability from short term sampling, for a variety of distributions [1]. Recent work supports the finding that PV is robust to rare events, showing lower standard error in PV compared to other metrics using jackknife estimates [13]. PV has now been applied to quantify variability in populations of a variety of species [14], [15], in oceanography [16], characterizing frequency distributions [17], and quantifying variability in backscatter measurements from acoustic surveys [18]. The present treatment provides an analytical basis for the further development of PV, and many additional characteristics and applications are likely to be discovered as it is implemented in various contexts by researchers with different expertise. Overall, our results indicate a strong case for the general adoption of PV as a standard measure of variability: it is a truly proportional first order measure of variability, avoids issues associated with standard techniques, and provides a robust common ground to summarize and compare variability in systems undergoing a wide variety of dynamic behaviour. We advocate PV as a useful common ground for evaluating and comparing stability (1/PV) and variation throughout the sciences. It is our intention to encourage critical thinking about philosophies of variability, and to raise skepticism and caution in applying and interpreting other approaches such as the Coefficient of Variation. Redefining our paradigm of variability will be particularly important for addressing contemporary issues of economic and climatic variability, and for establishing relationships between variability in physical, biological and socioeconomic quantities.
